# Biliary Radiofrequency Ablation and Sphincterotomy Restenosis: A Unique Case of Biliary Obstruction

**DOI:** 10.14309/crj.0000000000001824

**Published:** 2025-09-08

**Authors:** Muhammad Saad Faisal, Muneer Hasso, Abdulmalik Saleem, Muhammad Salman Faisal, Sumit Singla

**Affiliations:** 1 Department of Internal Medicine, Henry Ford Hospital, Detroit, MI; 2 Department of Gastroenterology and Hepatology, Henry Ford Hospital, Detroit, MI

**Keywords:** endoscopic retrograde cholangiopancreatography (ERCP), biliary radiofrequency ablation (RFA), cholestasis, ampullary adenocarcinoma, sphincterotomy restenosis

## Abstract

Biliary radiofrequency ablation is an emerging adjunctive and palliative therapy for patients with ampullary and biliary tumors. Given the high mortality for these malignancies, data on long-term complications are limited. We report a unique case of sphincterotomy restenosis causing biliary obstruction in a 98-year-old woman with a history of ampullary adenocarcinoma treated with papillectomy and biliary radiofrequency ablation (RFA). Endoscopic retrograde cholangiopancreatography revealed restenosis at the sphincterotomy site, managed successfully with repeat sphincterotomy and stenting. This case highlights sphincterotomy restenosis as a potential late complication of biliary RFA and emphasizes the need for awareness of delayed biliary obstruction in post-RFA patients.

## INTRODUCTION

Biliary radiofrequency ablation (RFA) is increasingly used as an adjunctive therapy for ampullary and biliary malignancies, offering local tumor control and palliation of obstructive jaundice. This technique involves endoscopic delivery of thermal energy through high-frequency current directly to biliary tumors, causing coagulative tissue necrosis. This is followed by stent placement to ensure biliary drainage^1^ While its clinical benefits in relieving cholestasis and as an adjunctive therapy for tumors is well recognized, there are very limited data regarding the complications and long-term effects of this procedure.^2^ Given the local application of thermal energy, one potential complication is sphincterotomy restenosis, a late consequence of biliary sphincterotomy that may contribute to recurrent biliary obstruction^3^ We present a case where biliary RFA contributes to sphincterotomy restenosis in a patient with a history of adenocarcinoma of the ampulla of Vater managed previously by papillectomy and RFA, underscoring the importance of recognizing this potential complication in post-RFA patients.

## CASE REPORT

**Figure 1.**
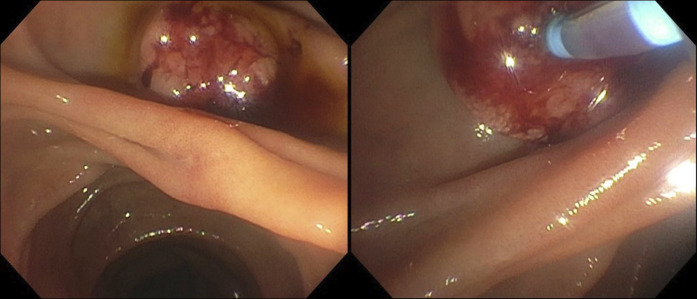
Ampullary mass before resection.

**Figure 2.**
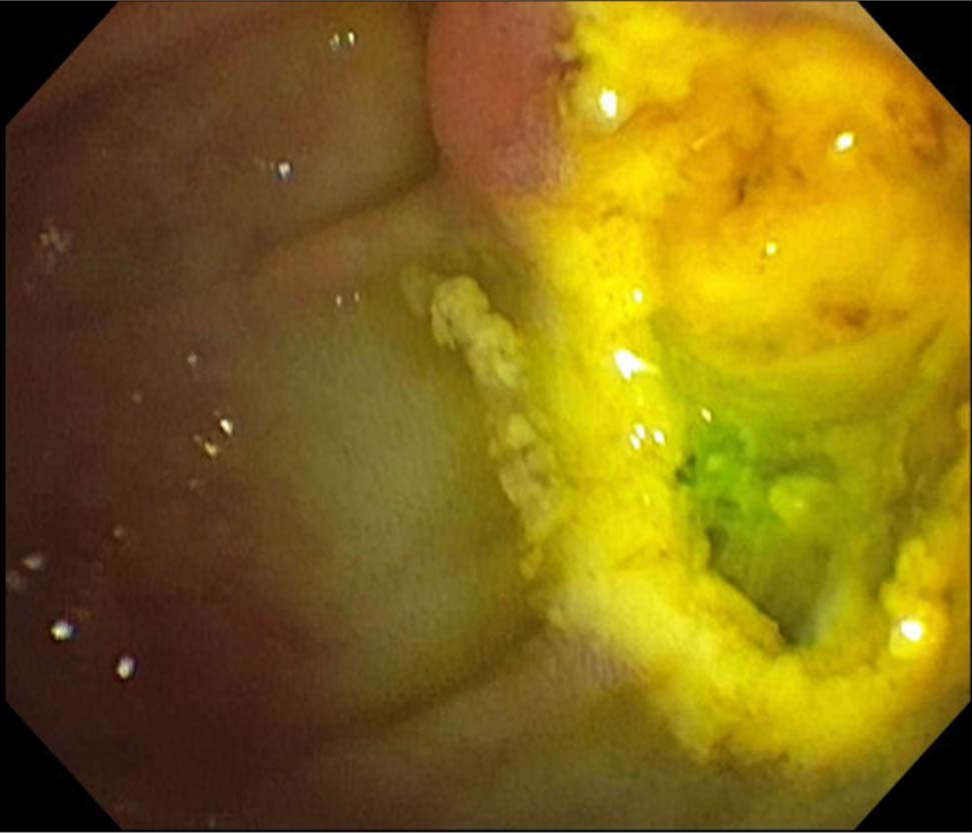
Ampullary mass after resection.

**Figure 3.**
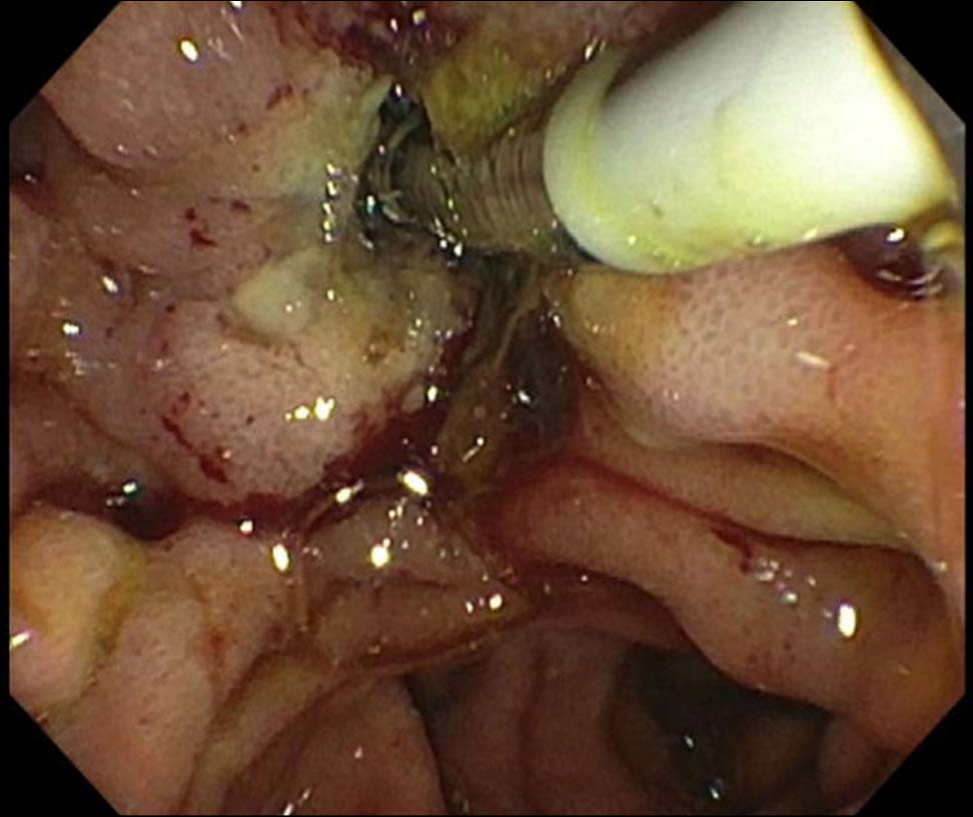
Endoscopic retrograde cholangiopancreatography with biliary radiofrequency ablation.

A 98-year-old woman with a personal history significant for adenocarcinoma of the Ampulla of Vater with short intraductal extension 4 years ago (Figure 1), managed successfully by papillectomy (Figure 2) and 1 cycle of biliary RFA (Figure 3) with negative surveillance, presented with a 2-week history of jaundice, nausea, and pruritis. Laboratory results were remarkable for cholestasis. Given the history of ampullary adenocarcinoma, an emergent endoscopic retrograde cholangiopancreatography was performed.

**Figure 4.**
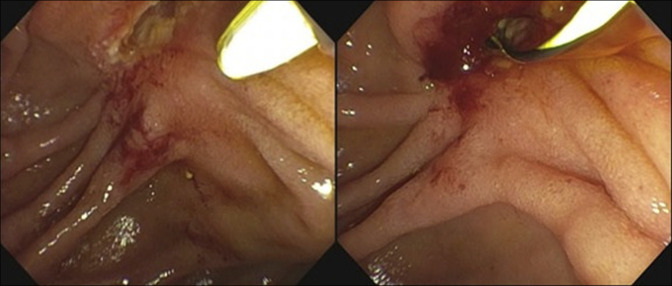
Patent sphincterotomy at last surveillance endoscopic retrograde cholangiopancreatography.

**Figure 5.**
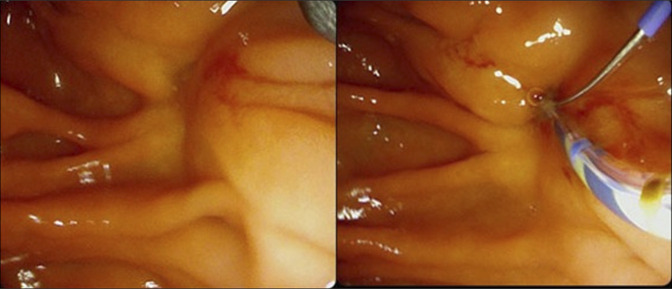
Sphincterotomy restenosis.

**Figure 6.**
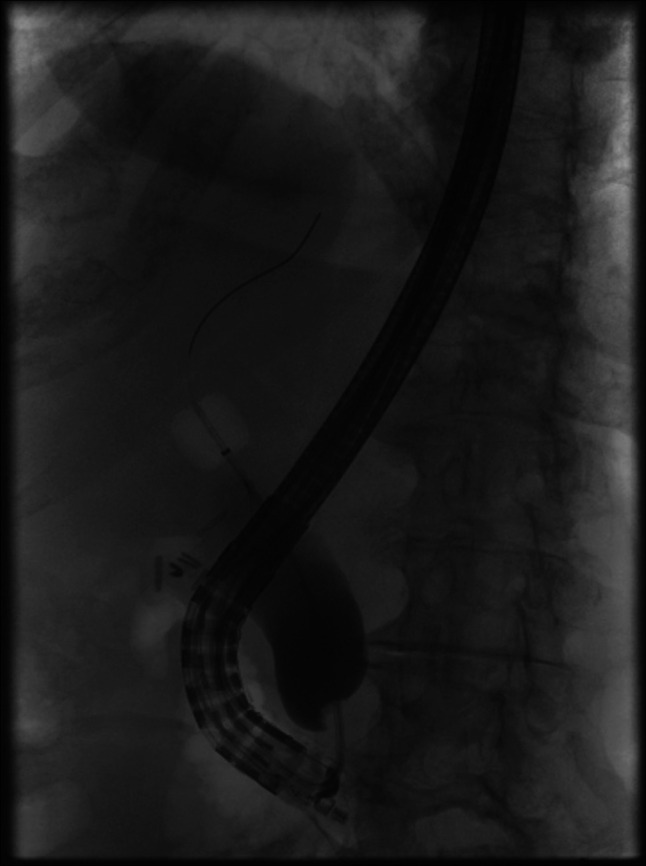
Fluoroscopic image at endoscopic retrograde cholangiopancreatography showing common bile duct dilation after patient presented with cholestasis.

Endoscopic retrograde cholangiopancreatography findings were significant for complete restenosis of the previously performed sphincterotomy, which was widely patent after the last surveillance 3 years ago (Figures 4 and 5). The bile duct was cannulated using a traction sphincterotome, and fluoroscopic images demonstrated marked and diffuse dilation of the common bile duct (CBD) up to 25 mm, along with severe stenosis at the pancreaticobiliary junction (Figure 6). Biliary sphincterotomy was subsequently performed using electrocautery with immediate release of sludge, purulence, and contrast, resulting in decompression of the CBD. This was followed by the placement of a fully covered metal stent with an anchoring double pigtail plastic stent in the CBD. Postprocedural fluoroscopic images did not show signs of strictures, stones, or dilation of the common bile duct. The patient had improvement in pruritis as well as liver enzymes after the procedure, and she was discharged home on the same day. Biopsies from the papilla and intra-ampullary biliary tree did not show recurrence of malignancy.

## DISCUSSION

Sphincterotomy restenosis is a documented late complication after biliary sphincterotomy, often resulting from scarring or fibrotic changes at the sphincterotomy site. Previous literature suggests that repeated biliary interventions, including sphincterotomy, can predispose patients to this complication.^3^ We present a unique case where biliary RFA contributed to sphincterotomy restenosis causing cholestasis.

RFA is a novel technique that is increasingly being used for the management of ampullary and biliary malignancies, both as an adjuvant therapy as well as with palliative intent to relieve biliary obstruction.^4^ Given the high mortality associated with biliary malignancies, long-term data and complications of this technique are not well studied.^5^ Our patient received RFA in the lower third of the CBD and developed sphincterotomy restenosis causing obstructive jaundice 3 years after receiving RFA.

Although there are limited data on the role of RFA in biliary stricture formation, data can be extrapolated from complications of RFA use in the esophagus. RFA is used for endoscopic ablation in the management of Barrett esophagus, with or without resection techniques such as endoscopic mucosal resection or endoscopic submucosal dissection.^4,6^ The principles used for Barrett esophagus are similar to those of biliary RFA, where locally applied radiofrequency delivers thermal energy, causing coagulative necrosis. Esophageal strictures are one of the most prevalent complications of esophageal RFA, with some studies reporting a stricture rate as high as 11.8% post RFA.^7,8^ Given that the physical stress to esophageal mucosa is similar to the stress on biliary tissue using RFA, we can extrapolate that RFA can increase the risk tissue growth leading to strictures and, in our case, sphincterotomy restenosis.

Given the paucity of data available regarding long-term complications of biliary RFA, our patient with sphincterotomy restenosis occurring years after biliary RFA is the first case in the literature to associate sphincterotomy restenosis to prior RFA. This case highlights the importance of maintaining a high index of suspicion for sphincterotomy restenosis in patients with a history of biliary RFA for ampullary lesions. In patients who present with signs of cholestasis or recurrent biliary obstruction after prior sphincterotomy, the possibility of restenosis should be considered along with recurrence of malignancy. Management involves repeat sphincterotomy with stent placement.

In this case, we demonstrate that biliary RFA, although an effective adjunct in the treatment of ampullary malignancy, may contribute to sphincterotomy restenosis and subsequent biliary obstruction. Clinicians should be aware of this potential complication, especially in patients with a history of complex biliary procedures. Furthermore, there is no available literature on prevention of restenosis. Continued research is needed to explore the risk factors associated with restenosis, mechanisms behind this phenomenon, and to evaluate potential strategies for preventing or managing restenosis in such patients.

## DISCLOSURES

Author contributions: MS Faisal, Muneer Hasso, A. Saleem: Conducted the literature review and wrote the initial manuscript draft. S. Singla, MS Faisal: Managed the patient, contributed to manuscript revision, provided clinical oversight, and approved the final version. M Saad Faisal is the article guarantor.

Financial disclosure: Dr. Sumit Singla is a consultant for Boston Scientific. All other authors declare no financial disclosures.

Informed consent was obtained for this case report.

## ABBREVIATIONS:

BE: Barrett’s Esophagus
CBD: common bile duct
EMR: endoscopic mucosal resection
ERCP: endoscopic retrograde cholangiopancreatography
ESD: endoscopic submucosal dissection
RFA: radiofrequency ablation
